# Gender Bias When Using Artificial Intelligence to Assess Anorexia Nervosa on Social Media: Data-Driven Study

**DOI:** 10.2196/45184

**Published:** 2023-06-08

**Authors:** David Solans Noguero, Diana Ramírez-Cifuentes, Esteban Andrés Ríssola, Ana Freire

**Affiliations:** 1 Telefonica I+D Telefónica Research Barcelona Spain; 2 Web Science and Social Computing group Universidad Pompeu Fabra Barcelona Spain; 3 Computer Vision Center Bellaterra (Cerdanyola del Vallès) Spain; 4 Departamento de Ciencias Básicas Universidad Nacional de Luján Luján Argentina; 5 Innovation and Sustainability Data Lab UPF Barcelona School of Management Barcelona Spain

**Keywords:** anorexia nervosa, gender bias, artificial intelligence, social media

## Abstract

**Background:**

Social media sites are becoming an increasingly important source of information about mental health disorders. Among them, eating disorders are complex psychological problems that involve unhealthy eating habits. In particular, there is evidence showing that signs and symptoms of anorexia nervosa can be traced in social media platforms. Knowing that input data biases tend to be amplified by artificial intelligence algorithms and, in particular, machine learning, these methods should be revised to mitigate biased discrimination in such important domains.

**Objective:**

The main goal of this study was to detect and analyze the performance disparities across genders in algorithms trained for the detection of anorexia nervosa on social media posts. We used a collection of automated predictors trained on a data set in Spanish containing cases of 177 users that showed signs of anorexia (471,262 tweets) and 326 control cases (910,967 tweets).

**Methods:**

We first inspected the predictive performance differences between the algorithms for male and female users. Once biases were detected, we applied a feature-level bias characterization to evaluate the source of such biases and performed a comparative analysis of such features and those that are relevant for clinicians. Finally, we showcased different bias mitigation strategies to develop fairer automated classifiers, particularly for risk assessment in sensitive domains.

**Results:**

Our results revealed concerning predictive performance differences, with substantially higher false negative rates (FNRs) for female samples (FNR=0.082) compared with male samples (FNR=0.005). The findings show that biological processes and suicide risk factors were relevant for classifying positive male cases, whereas age, emotions, and personal concerns were more relevant for female cases. We also proposed techniques for bias mitigation, and we could see that, even though disparities can be mitigated, they cannot be eliminated.

**Conclusions:**

We concluded that more attention should be paid to the assessment of biases in automated methods dedicated to the detection of mental health issues. This is particularly relevant before the deployment of systems that are thought to assist clinicians, especially considering that the outputs of such systems can have an impact on the diagnosis of people at risk.

## Introduction

### Background

In recent years, we have witnessed unprecedented improvements in the automation of a broad range of processes that affect our everyday lives, owing to the application of artificial intelligence (AI) and, in particular, the use of machine learning (ML). Self-driving car navigation [1], medical imaging and diagnostics [2], speech recognition [3], and recommender systems [4] are just a few examples of cutting-edge technologies that show the potential that ML has to increase the quality of our lives. Particularly in the domain of mental and behavioral health, there has been increasing research interest in the identification of mental health state alterations through the exploitation of web-based digital traces. On a daily basis, many people are increasingly using social media platforms to share their feelings and moods. This creates a unique opportunity to proactively identify linguistic patterns that correlate with mental disorders [5,6]. Early risk prediction of depression and anorexia [7] and suicide risk assessment [8,9] are just some examples of different initiatives that have fostered research on the interaction between language and mental health disorders on web-based social media and the application of ML to address such challenges.

However, as ML becomes more pervasive in sensitive domains, special care should be paid to a recent issue that has drawn scholars’ attention: *algorithmic bias*. The great success of ML algorithms resides in their ability to indiscriminately learn latent nuances in the input data even if they are not explicitly instructed to do so. However, human data encode human biases by default [10], and therefore, these algorithms are prone to replicate and even amplify such biases in their outcomes, leading to *unfair* decisions.

In the context of risk assessment and decision-making systems, fairness is defined as the “absence of any prejudice or favoritism toward an individual or a group based on their inherent or acquired characteristics” [11]. Hence, an algorithm whose decisions are skewed toward a particular group of people, often a certain minority group, is considered *unfair*. In recent years, several cases have been identified as examples of inequalities created or amplified by AI-based systems. Such systems are trained using data extracted from society, often reflecting several stereotypes in the form of biases. Examples of how data biases are then reflected in the predictions of automated systems can be found in the case of Correctional Offender Management Profiling for Alternative Sanctions (COMPAS), a recidivism prediction tool used in the United States, where ProPublica identified a much higher false positive rate for Black people [12]; XING, a job platform that was reported to rank less qualified male candidates higher than more qualified female candidates [13]; or face recognition web-based services found to achieve much lower accuracy on female individuals with darker skin color [14]. When dealing with health-related data, these biases can be particularly harmful. As argued by Walsh et al [15], health disparities contribute to algorithmic bias. For example, women have a higher prevalence of major depressive and anxiety disorders [16]. Prevailing societal notions about several groups’ susceptibility to mental disorders contribute to incorporating bias into the underlying data and model specification. Furthermore, this issue, along with other factors, might prevent most of the risk assessment and decision-making technological developments from ever being used in real-life settings [15].

In this study, we performed an exploratory analysis that considered algorithmic fairness when characterizing eating disorders on social networks. In particular, we studied a classification problem in which an automated system predicted, given a set of posts authored by a user on a social media platform, whether such a person might have an eating disorder (positive prediction) or not (negative prediction). We observed, quantified, and characterized different types of inequalities that led to an unfair predictive system. To the best of our knowledge, this is the first study in which algorithmic fairness was applied to the detection of eating disorders on social networks. This contribution focused on how inequalities can be easily created or amplified by predictive systems in the domain of eating disorders. In addition, we used state-of-the-art techniques to mitigate detected biases and show that finding a solution that removes all existing biases is a very arduous task.

The remainder of this paper is structured as follows: the *RQs or Objectives* and *State of the Art* sections state the research questions (RQs) and the previous work in this regard; the *Methods* section describes the proposed methods for bias detection, characterization, and mitigation; in the *Results* section, we discuss the main results found; and the *Discussion* section closes the paper with additional discussion pointing to the limitations of this research.

### RQs or Objectives

In this context, we defined the following RQs that guided our experimental setup, analysis, and contributions: (1) *To what extent* *ML-based predictive models exhibit performance disparities across anorexia nervosa (AN) demographic groups?* (RQ 1), (2) *What are the causes of the existing biases when assessing AN on social media using ML algorithms?* (RQ 2), and (3) *How can we mitigate the aforementioned biases?* (RQ 3).

### State of the Art

#### Characterization and Assessment of Mental Disorders on Social Media

Traditionally, mental health practitioners have collected and integrated information from various instruments to characterize the mental state of individuals [17]. These include direct observation, focused questions on current symptoms, and formalized psychological tests. Such instruments have been used to assess several mental health–related variables, such as the appearance, mood, and attitudes of subjects, to determine the presence of any irregularity. The proliferation of web-based social media platforms is changing the dynamics in which mental health state assessments are performed [18-20]. Individuals are using these platforms on a daily basis to share their thoughts, as well as to disclose their feelings and moods [21,22]. As such, these sites have become promising means to detect different mental health disorders as the language used as well as the emotions expressed in the text (eg, social media posts), shared with followers or friends on a daily basis, may pinpoint feelings such as worthlessness, guilt, or helplessness [23-25]. This can provide a characterization of the symptoms of psychological disorders such as AN. In this regard, the study by Ramírez-Cifuentes et al [5] characterized different stages of AN on Spanish-speaking Twitter users by combining the analysis of text, images, and social interactions.

#### Algorithmic Fairness for Detecting Mental Health Status

Limited research has been conducted regarding the intersection between algorithmic fairness and the automated detection of mental disorders. However, this is of increasing interest, especially for social media platforms or in scenarios in which users give their consent to be tracked on social media for health monitoring (schools or medical centers). In particular, the study by Chancellor et al [26] highlights the existence of methodological concerns regarding data collection processes and bias related to the application of ML methods to infer mental health status. In general, discussions of consent, validity, underlying bias from data collection techniques, and ML model selection are very limited. Moreover, the outcome of such algorithms, which perpetuate unintended biases, might lead to negative and discriminatory repercussions.

In this respect, Straw and Callison-Burch [27] conducted a literature review of 52 articles that addressed the use of natural language processing in mental health across multiple disciplinary databases and explored each stage of AI model development to analyze which and how biases arise. The literature review found that no studies stratified the outputs of their natural language processing models by demographic features. Moreover, they performed an analysis of biases in word embeddings that relate to mental health by comparing demographic labels and psychiatric terms (eg, man is to depression as woman is to *perinatal_depression*). They evaluated Global Vector for Word Representation (GloVe) and Word2Vec pretrained embeddings. Word embeddings allow for the capture of the meaning of words by means of linear representations in a high-dimensional semantic space. Thus, the semantic content of a word is encoded as a vector, and this vectorial representation can be used to estimate how semantically close other words are.

More recently, the study by Aguirre et al [28] explored the susceptibility to gender and racial biases of different computational methods for the automatic assessment of depression. In particular, they focused on the detection and mitigation of such demographic biases analyzing 2 widely used data sets for the study of depression on social media: CLPsych [29] and MULTITASK [30]. They considered 4 demographic groups and 2 genders. The outcomes of their study revealed that existing data sets are not demographically representative and, without accounting for this, depression classifiers performed worse on people of color, specifically female individuals in CLPysch and male individuals in MULTITASK. Both groups were underrepresented in the data sets. Finally, they provided a series of recommendations on how to avoid such biases in future research using these data sets.

Prior work differs from ours in that we present the analysis and characterization of gender-related biases regarding a particular use case, the detection of AN on Twitter. For our predictive models, we considered several features and proposed strategies to address biases by applying fairness assessment approaches.

## Methods

### Data Set

We used the data set collected by Ramírez-Cifuentes et al [5] for characterizing AN on social media, in particular on Twitter. This data set consists of publications in Spanish corresponding to a 1-year period between December 21, 2017, and December 21, 2018. The metadata elements and texts extracted passed through a strict transformation process to build and store vector representations of the features of interest at the user level, guaranteeing the analysis of fully anonymized data.

As stated in the study by Ramírez-Cifuentes et al [5], the data set consists of Spanish-language tweets related to eating disorders on Twitter. To create this collection, researchers manually collected and classified keywords and popular hashtags commonly used by eating disorder communities; phrases likely to be used by people undergoing treatment; and terms used by recovered users from multiple sources, including proanorexia blogs, academic publications, and documents from the Spanish Association Against Anorexia and Bulimia. In addition, a survey was conducted among volunteers who had recovered from AN to evaluate and filter the collected phrases and keywords. The study collected 114,627 public tweets containing the search phrases, and 645 users were selected for labeling purposes. They were classified by a group of psychologists, psychiatrists, and therapists into one of the following groups: AN users, treatment users, recovered users, a focused control group, a random control group, and doubtful cases (later discarded). The focused control group consisted of users collected at first who made use of keywords related to anorexia but who were labeled as control cases during the annotation phase. In contrast, the random control group consisted of a random sample of Twitter users selected using Twitter’s Sample Tweets application programming interface; annotators made sure that no users with AN were part of this group.

In addition, to ensure the protection of users’ privacy and identity, generic identifiers were assigned to both the users and their posts, and all personal information was removed from their descriptions and tweet texts, including usernames, proper nouns, URLs, email addresses, location names, and numbers. The extracted metadata elements and texts underwent a strict transformation process to create vector representations of the features of interest, which enabled the analysis of fully anonymized information. To label the tweets, only users who had at least 3 tweets containing the selected keywords in each category were considered. Before submitting the text samples to annotators, the tweets’ texts were anonymized and translated into English to prevent users from being reidentified based on their writings. Features were extracted as data were being collected, and no one was able to read the actual texts. Only the extracted transformed features were stored [5].

For this study, the groups of the data set were assigned to the following categories: (1) *Anorexia Nervosa (positive)*—177 users (471,262 tweets) who manifested signs and symptoms of AN in their texts or explicitly stated that they had been diagnosed with AN or were in treatment (AN + treatment users), including users at the precontemplation, contemplation, and treatment stages according to the transtheoretical model [31]—and (2) *control*—326 users (910,967 tweets) who did not make use of terms related to AN or users who used terms related to the disorder but did not manifest signs of anorexia (focused control + random control). Table 1 shows the number of positive and control cases split by gender. We considered only male and female users, discarding those users corresponding to organizations that were also included in the original data set. We also discarded users with missing data.

The age and gender of Twitter users are not publicly displayed, so a method for demographic inference was used in the study by Ramírez-Cifuentes et al [5]. This involved using a deep neural architecture developed by Wang et al [32] to classify the age, gender, and organizational status of social media users. The model was trained using data in 32 languages, including Spanish, and analyzed the users’ names, profile descriptions, and pictures. This method was implemented using the M3-Inference Python library (Python Software Foundation). The approach was evaluated on a group of manually labeled users and achieved a macroaverage accuracy of 0.84 for all gender groups and 0.80 for all age groups.

**Table 1:** Base rates for each class and gender in the data set.

	Positive (n=177), n (%)	Control (n=326), n (%)
Female	127 (71.8)	157 (48.2)
Male	50 (28.2)	169 (51.8)

### Feature Description

The data set included >100 features built and inferred based on the text, images, and metadata of the users’ tweets. We discarded features extracted from images as they were not present in all the users because most of them tweeted text with no images attached. A detailed description of all the features included in the data set can be found in the data set paper [5], and the features can be clustered into 4 groups, as described in Textbox 1. These features were extracted considering various perspectives, such as language, psychology, relationships, behavior, demographics, and visual aspects.

According to Ramírez-Cifuentes et al [5], the textual content shared by users on Twitter is analyzed based on linguistic and psychological aspects grouped into 6 categories. Some of these categories are based on a classification given by the Linguistic Inquiry and Word Count 2007 Spanish version, which categorizes words into psychologically meaningful categories. The remaining categories were defined considering the psychological aspects related to eating disorders stated by eating disorder experts. The categories analyzed were linguistic dimensions (24 features), vocabulary related to risk factors (10 features), vocabulary related to anorexia (9 features), and user interests (200 topics).

The linguistic dimension features are based on the use of grammatical and syntactical elements such as pronouns, verbs, adverbs, prepositions, and articles considering different tenses and types of pronouns. In total, 24 linguistic dimension characteristics were explored, and many of them could distinguish AN users from both control groups. It was found that the use of first-person singular pronouns, along with a high use of negations and a reduced use of articles, characterized AN users’ posts.

The affective and emotional processes were analyzed using Linguistic Inquiry and Word Count and EmoLex dictionaries, which associate words with 8 basic emotions and 2 sentiments. A sentiment analysis tool called Senti-py was also used to provide a polarity value for an individual text. In total, 29 affective and emotional process characteristics were explored.

Personal concerns and biological processes, vocabulary related to risk factors, vocabulary related to anorexia, and user interests were the remaining categories analyzed, each with 12, 10, 9, and 200 characteristics, respectively. These categories aimed to capture the psychological aspects related to eating disorders and user interests.

The analysis also focused on demographic features, which include age and gender groups, and the social network of users, including measures of interaction, popularity, and support received by users through social media. The following features were extracted and calculated for each user: number of followers, number of users they followed, number of total favorites given to the publications of other users, average number of favorites received by the user, and average number of publications shared by other users.

Finally, in the analysis of behavioral aspects, features were extracted to explore elements that may link the frequency of social media use with AN. An example is the level of activity of users during the night, which could be an indicator of insomnia, a sign that has been linked to related disorders such as suicidal ideation. These characteristics were extracted from the metadata of tweets, and the behavior of users was measured based on their daily, weekly, and monthly activity.

In the following sections, we describe the methodology used to detect and quantify biases in models trained on the AN data set to answer the RQs posed in the *Introduction* section.

**Types of features included in the selected data set.**

**Content shared and interests**

- Linguistic dimensions
- Affective processes and emotions
- Personal concerns
- Risk factor vocabulary
- Anorexia-related vocabulary
- Topics of interest
- Proportion of anorexia nervosa–related tweets

**Social network**

- Measures of interactions and engagement
- Analysis of followees and community detection
- Analysis of interests between users and their followees

**Behavioral aspects**

- Activity on a daily, weekly, and monthly basis
- Sleep period tweeting ratio

**Demographics**

- Gender
- Age

### Bias Detection (RQ 1)

To answer RQ 1, we evaluated two scenarios: (1) the first scenario corresponds to the most typical case, when a unique model is trained for both genders and used to make predictions for all samples, and (2) in the second scenario, we trained an individual model for each gender. We used this approach to evaluate whether this might have a substantial impact on the final results.

To show that the observed behavior does not specifically depend on the use of a certain category of classifiers, we compared a variety of models commonly used for the task [20]: (1) logistic regression, (2) random forest, (3) support vector machines (SVMs) with different kernels, (4) multilayer perceptron (MLP), and (5) AdaBoost.

To test the models, we partitioned the data set between training and testing using a cross-validation strategy based on 5 folds. For each of these data partitions, we trained a classifier using the training set and evaluated the observed performance on the testing set.

The proposed methodology allows for the generalization of results on multiple data partitions and different models.

Biases were measured in terms of balanced accuracy (bAcc) and false negative rate (FNR) ratios between samples of different genders. FNR is related to the criteria of sufficiency [33] and requires a fair model to have similar FNRs across demographic groups. The bAcc metric is generally preferable in scenarios where data are not well balanced, as is the case in the collected data set.

*bAcc* normalizes the true positive rate, also known as *recall*, and true negative rate predictions by the number of positive and negative samples, respectively, and divides their sum by 2. The true positive rate and true negative rate measure, respectively, the fraction of correctly detected positives and negatives among their total number:



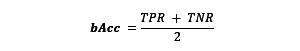



The *FNR* quantifies the fraction of false negatives among the number of positives:



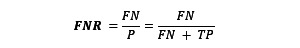



Finally, we measured the following (values closer to 1 indicate less biased predictions):



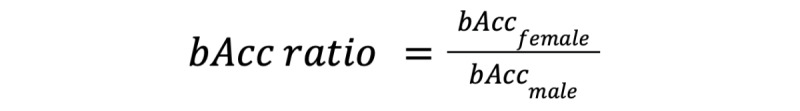





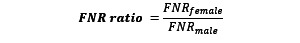



### Bias Characterization (RQ 2)

To answer RQ 2 (investigating the causes of the algorithmic bias when assessing AN on social media), we studied the features considered as input for the predictive models to identify which of those variables are more predictive for each gender (Textbox 1).

We separated the instances by gender and proceeded to apply feature selection approaches. In particular, we considered recursive feature elimination (RFE) [34] to analyze the relevance of features depending on the gender of the users. RFE starts with all features, and then a subset of *k* features (the most relevant) is searched by removing features until the desired number remains. It works by training an estimator on the initial set of features; then, features are ranked by importance based on the estimator. Afterward, features that are less important are removed sequentially from the current set of features so that the process can be recursively repeated on the pruned set until the number *k* of desired features to keep is reached. In our case, we used a logistic regression estimator and obtained a rank for all the features used by assigning a value of 1 to *k* as it provides a rank based on the order in which features were removed at each iteration until only 1 feature was left. We used the Python sklearn RFE feature selection implementation [35]. Considering the top 10 (ie, *k*=10) features selected through this approach for each gender model, we made comparative plots of their distributions to observe how the values of the selected features differed.

To investigate whether the models selected the same features as a group of real experts on eating disorders, we asked 5 clinicians to answer a survey. These clinicians were experts who had participated in social media writing labeling tasks. They were asked to assign a level of importance to the different feature types extracted from the data set (considering that they should predict AN risk just based on writings, as our models did). These feature types explore the use of grammatical and syntactical elements and the use of terms related to emotions, personal concerns, social support received, biological processes and health, suicide risk factors, and eating disorder–related vocabulary. We also considered behavioral patterns that implied a prolonged use of social media and demographic elements such as age and gender.

The importance levels ranged from 1 to 5, where assigning a score of 1 meant that the feature type was not relevant, whereas a score of 5 meant that the feature type was very important for the screening of AN. Clinicians were allowed to add comments regarding the feature types suggested. Later, we calculated the means, medians, and SDs of the scores assigned to each feature type and applied different approaches to measure the interrater agreement.

On the basis of the expert assessment results, we proceeded to compare their feature type importance with the relevance assigned by a predictive model trained on all the instances and features. We used the RFE rank of the generic model and assigned a score equivalent to its inverse rank position to each feature, meaning that the feature ranked first obtained a score equivalent to the rank of the last feature in the ranking. This score corresponded to the importance level assigned to the feature based on an automated predictive model. Later, each feature was mapped to the feature type to which it belonged to average the scores obtained by all the features belonging to a given feature type. Once a single score was obtained for every feature type, we proceeded to compare the scores obtained by the classifier with those assigned by the experts. A normalization process was applied before to scale the scores of each group (model and experts) between 0 and 1.

Note that the proportion of AN-related tweet features is given by a deep learning classifier [5] that takes word embeddings as input (vector representations of the terms found in the users’ writings). Taking into account this aspect, we considered the features within the anorexia-related vocabulary feature type.

### Bias Mitigation (RQ 3)

In this section, we assess the effect of state-of-the-art bias mitigation algorithms applied to the use case studied in this work to answer RQ 3.

#### Training Fair Classifiers

Existing methods to mitigate biases in ML models fall under 3 categories [11] (Textbox 2).

In particular, we used as baseline a logistic regression model, identified as the model with a better trade-off between bAcc and FNR ratio. Such a baseline was compared with the effects of applying 2 preprocessing algorithms named *optimized preprocessing* [36]—with a repair level of 0.85—and reweighting [37]. In addition, we tested a postprocessing algorithm named *calibrated equalized odds*.

**The 3 categories of bias mitigation methods in machine learning (ML) models.**

**Preprocessing**

- Preprocessing methods modify the input data with the objective of reducing input data biases that might lead to performance disparities.

**In-processing**

- In-processing techniques modify the learning algorithm to incorporate fairness constraints.

**Postprocessing**

- Postprocessing approaches treat the ML model as a black box and modify its outputs to achieve fairer outcomes.

#### Training Calibrated Classifiers

Previous work [38] has analyzed the trade-off between minimizing error disparities across population groups and maintaining calibrated probability estimates. Obtaining calibrated probability estimates is considered crucial for empirical risk analysis tools [39].

Model calibration is often considered in algorithmic fairness analysis, as in the case where, if there is a disparity in calibration between population groups, a decision maker may be inclined to take the predictions less seriously for the group that lacks calibration [40].

When the classifier predictions are properly calibrated, its output can be directly used as a probability. It requires that, for each classifier output range, the proportion of samples that actually have the true label be equivalent to the output value. For example, if a given (binary) classifier is properly calibrated, a prediction score of 0.2 for a given sample would require it to have a 20% chance of belonging to the positive class, a prediction score of 0.5 would require it to have a 50% chance of belonging to the positive class, and so forth.

In the task of detecting AN from web-based traces, certain use cases such as giving treatment priority to higher-risk cases would also require the use of a continuous output, that is, predicting values in the range (0,1) so that those predicted with higher values can be used to prioritize treatment for those cases that are at a higher risk or have a higher probability of having the disorder.

In addition, comparing calibration across demographic groups can be used to adapt the decision threshold individually for each demographic group so that the conditional probabilities of obtaining false negatives are equalized between them.

To obtain calibrated classifiers, we compared the performance of the state-of-the-art isotonic and sigmoid calibrators (we used the implementation available in scikit-learn [41]), which can be understood as regressors that map input values to new projected values in the same range (0,1), forming a new distribution where the obtained scores are equivalent to the actual chances of being a positive sample.

To train each calibrator, we used 5-fold cross-validation to ensure the correct generalization of the obtained results. For each data split, the predictions of the trained model were used to fit an instance of each calibrator. To obtain calibrated predictions, model predictions were then transformed into the average of the 5 trained calibrators.

### Ethical Considerations

Research involving human beings concerns sensitive topics related to the ethics of the treatment of data and individuals’ privacy [26]. The sensitive nature of mental health research requires us to consider the possible benefits of this study alongside its potential harms.

The potential immediate benefit of this study is a better understanding of gender bias in the computational assessment of AN using social media data. A potential second benefit is the mitigation of the disparities observed, which otherwise, as shown in this study, permeate into the assessment algorithms. In particular, we ascertained the extent to which fairer classifiers can be developed considering the trade-off with performance.

Nonetheless, we are aware of the potential harms of our work. Mental health status is a sensitive personal attribute that could be used to maliciously target individuals on publicly facing web-based platforms. Hence, as researchers working with social media data, we took the necessary precautions to protect the privacy of individuals and their ethical rights to avoid any further psychological distress. We followed the guidelines of Benton et al [42] and Ayers et al [43] on data use, storage, and distribution. All the analyses were conducted on deidentified versions of the data by altering (obfuscating) all identifying metadata to preserve the privacy of individuals in the data set.

## Results

In this section, we aim to answer RQ 1, RQ 2, and RQ 3, following the methodology described in the previous section.

### Bias Detection (RQ 1)

To know whether ML-based predictive models exhibit performance disparities across AN demographic groups (RQ 1), we trained and evaluated different estimators for assessing the risk of AN in male and female samples. Performance was measured using FNR and bAcc ratios. As stated in the previous sections, we compared two different scenarios: (1) a single model trained for both genders and (2) an individual model trained for each gender separately.

When analyzing scenario 1, the results on performance disparities showed that the trained models obtained a good level of bAcc (all of them >87%) and a low level of FNR (between 4% and 6%), with AdaBoost and random forest models reaching 92% accuracy. The SVM linear kernel produced the lowest accuracy result, and the SVM with the radial basis kernel showed the highest number of false negatives.

Nevertheless, we observed important performance differences when decomposing such values by gender, yielding in the worst case (MLP classifier, 1 model per gender) an accuracy of 0.913 for male samples and 0.844 for female samples (relative difference of 8% accuracy). This negative difference in accuracy for female samples was consistent for all the models.

**Figure 1:**
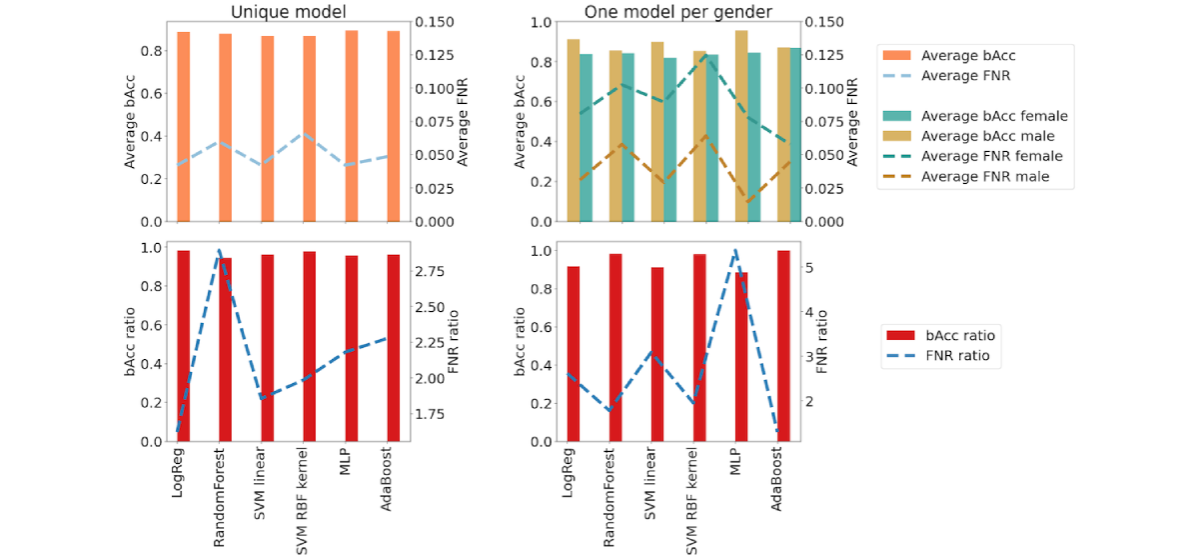
Average balanced accuracy (bAcc) and false negative rate (FNR) compared with bAcc ratio and FNR ratio across genders on the trained models. Figures on the left column represent scenario 1—unique model—and figures on the right column represent scenario 2—one model per gender. LogReg: logistic regression; MLP: multilayer perceptron; RBF: Radial Basis Function; SVM: support vector machine.

In addition, most of the models showed approximately twice the FNRs for female samples when compared with male samples (dotted blue line in Figure 1). The differences in performance were even more dramatic in the second scenario, where an individual model was trained for each gender, increasing the FNR differences by up to 500% in the case of the MLP model.

Although applying the second scenario might imply a disparate treatment by gender, which is protected by law in multiple countries, it was an interesting exercise that produced mostly counterintuitive results. The comparison of the results shown in Figure 1 also proves that including male samples in the training set benefited the performance obtained for female samples.

Summarizing the results obtained, we were able to achieve high-accuracy models in both scenarios, but the performance was always lower for female samples. An error analysis points out that female samples had higher rates of false negatives, which is extremely dangerous in this context as a false negative could lead to a lack of detection and, therefore, a denial of treatment. Disparities in performance were reduced when a unique model was trained for both genders. Conversely, using a different model per gender led to higher disparities in performance, increasing even more the lack of performance for female samples.

In the remainder of this paper, we use the logistic regression classifier with a unique model for both genders as a baseline. This choice was motivated by the fact that such a classifier shows the best bAcc and FNR ratios while maintaining an average accuracy of >87%.

These results motivated the remainder of this paper. As observed, female samples not only obtained a lower performance, but also, when the models made a misprediction, it was almost twice as likely that it had the form of a false negative for female samples.

### Bias Characterization (RQ 2)

**Figure 2:**
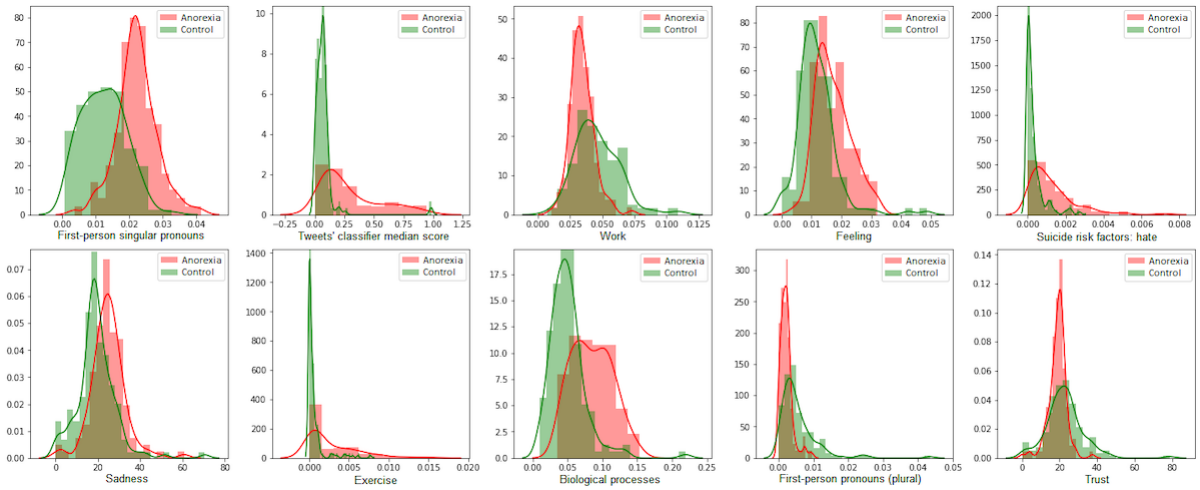
Top 10 features selected by the female data model.

**Figure 3:**
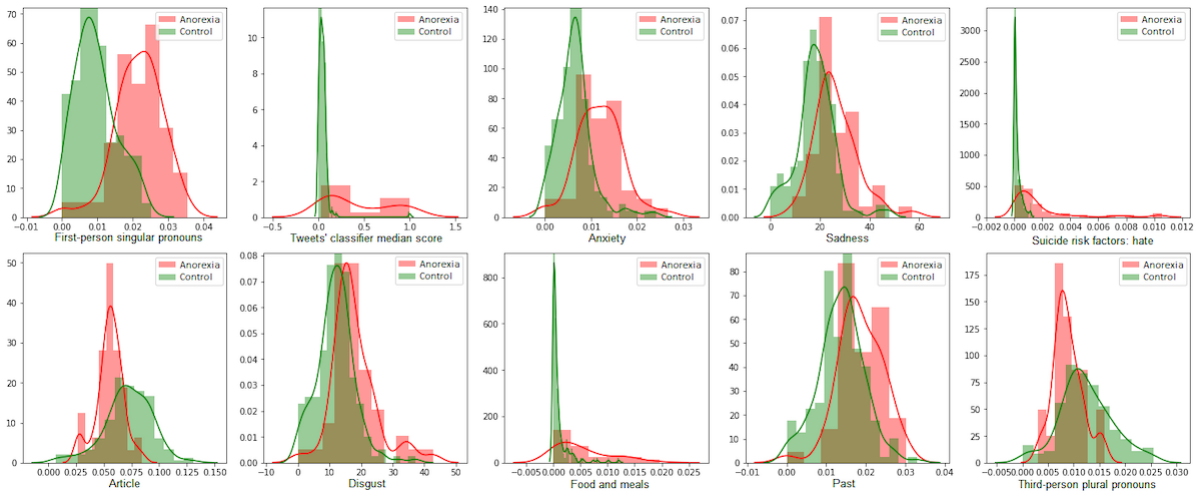
Top 10 features selected by the male data model.

Table 2 shows the top 10 features selected according to the RFE approach for each gender model using a logistic regression estimator for both cases. We also show the top 10 features given by a model (“Generic model”) with all instances (male and female samples) using gender as a feature. We can see that, for all the models, the most relevant features measured the use of first-person singular pronouns and the proportion of AN-related tweets. “Hate” as a suicide risk factor and “sadness” were features that were also important for all the models. The distribution of the top 10 features for the female and male models is shown in Figures 2 and 3.

**Figure 4:**
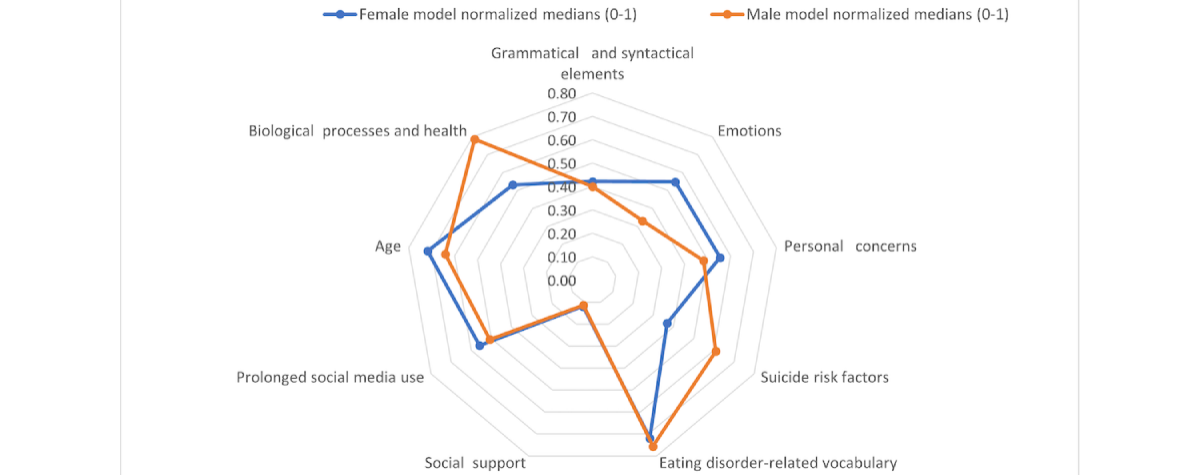
Feature importance for each gender model.

Figure 4 shows a comparison of the importance of each feature type for each gender model. To calculate the values, we followed the process described in the *Bias Detection (RQ 1)* section in the *Methods* section (predictive models vs clinicians). We noticed that eating disorder–related vocabulary was the most relevant for both, whereas biological processes and suicide risk factors were the most relevant for male samples and age, emotions, and personal concerns were the most relevant for female samples.

Table 3 shows the results of the survey administered to clinicians to determine the most important features they considered when assessing AN based on writings. We averaged the relevance scores assigned by the clinicians participating in the survey. Considering each question as a case and our 5 annotators as raters, we used 2 interrater agreement measures suitable for studies with >2 raters: the Fleiss κ (0.20) [44] and the intraclass correlation coefficient (ICC; 0.87) [45]. Of these measures, the ICC is one of the most commonly used statistics for assessing interrater reliability for ordinal variables [46]. The ICC results, which are more suitable for ordinal data, suggested good reliability, whereas κ indicated a slight agreement.

We also calculated the percentage of agreement [47] for multiple raters, and the individual agreement for each feature type is described in Table 3. The average percentage of agreement was 44%, which implies a moderate agreement.

The feature types that raters found most relevant were those that measured the use of eating disorder–related vocabulary, with full agreement among clinicians, along with suicide risk factors, biological processes and health, and gender. The least relevant feature type was related to the use of grammatical and syntactical elements.

The survey also asked for factors that are considered by clinicians in a medical consultation for AN screening. In this case, experts mentioned aspects such as weight, height, restrictive behaviors, obsessive personality, purgative behaviors, BMI, fear of gaining weight, daily life issues (work, school, and personal relationships), family members with a history of eating disorders, different physical indicators (thermoregulation difficulties and bradycardia), low self-esteem, and gender as women are more likely to be diagnosed with this type of eating disorder.

**Table 2:** Top 10 features selected according to the recursive feature elimination approach for each gender model.

Rank	Female model	Feature type	Male model	Feature type	Generic model	Feature type
1	First-person singular pronouns^a^	Grammatical and syntactical elements	First-person singular pronouns^a^	Grammatical and syntactical elements	First-person singular pronouns^a^	Grammatical and syntactical elements
2	Proportion of AN^b^-related tweets^a^	Proportion of AN-related tweets (implies the use of AN-related vocabulary)	Proportion of AN-related tweets^a^	Proportion of AN-related tweets (implies the use of AN-related vocabulary)	Proportion of AN-related tweets^a^	Proportion of AN-related tweets (implies the use of AN-related vocabulary)
3	Work	Personal concerns	Anxiety	Affective processes and emotions	Sadness^a^	Affective processes and emotions
4	Feeling	Affective processes and emotions	Sadness^a^	Affective processes and emotions	Suicide risk factors: hate^a^	Suicide risk factors
5	Suicide risk factors: hate^a^	Suicide risk factors	Suicide risk factors: hate^a^	Suicide risk factors	Articles	Grammatical and syntactical elements
6	Sadness^a^	Affective processes and emotions	Articles	Grammatical and syntactical elements	Biological processes	Biological processes and health
7	Exercise	Anorexia-related vocabulary	Disgust	Affective processes and emotions	Negative emotions	Affective processes and emotions
8	Biological processes	Biological processes and health	Food and meals	Anorexia-related vocabulary	Food and meals	Anorexia-related vocabulary
9	First-person pronouns (plural)	Grammatical and syntactical elements	Past	Grammatical and syntactical elements	Past	Grammatical and syntactical elements
10	Trust	Affective processes and emotions	Third-person pronouns (plural)	Grammatical and syntactical elements	Suicide risk factors: self-loathing	Suicide risk factors

^a^Features that are relevant for both models.

^b^AN: anorexia nervosa.

**Table 3:** Results of the survey answered by clinicians on the most important features for assessing anorexia nervosa based on writings.

Feature type	Description	Relevance (0-5), mean (SD)	Mode (0-5)	Median (0-5)	Agreement, %
Grammatical and syntactical elements	Use of grammatical and syntactical elements such as personal pronouns and verbs	1.60 (1.20)	1	1	60
Emotions	Use of terms related to emotions, such as “joy,” “sadness,” and “fear”	3.60 (0.49)	4	4	40
Personal concerns	Use of terms related to personal concerns, such as “work,” “leisure,” and “religion”	3.00 (1.26)	3	3	30
Social support	Use of terms related to social support, such as “friends,” “family,” and “loneliness”	3.60 (0.49)	4	4	40
Biological processes and health	Use of terms related to biological processes and health, such as “eating,” “therapy,” and “healing”	4.20 (0.75)	5	4	20
Suicide risk factors	Use of terms related to suicide risk factors, such as “self-harm,” “bullying,” and “substance abuse”	4.60 (0.49)	5	5	40
Eating disorder–related vocabulary	Use of terms related to eating disorders, such as “laxative names” and “weight concerns”	5.00 (0.00)	5	5	100
Prolonged social media use	Posting frequency	4.40 (0.49)	4	4	40
Age	User age	4.00 (0.63)	4	4	30
Gender	User gender	4.60 (0.49)	5	5	40

**Figure 5:**
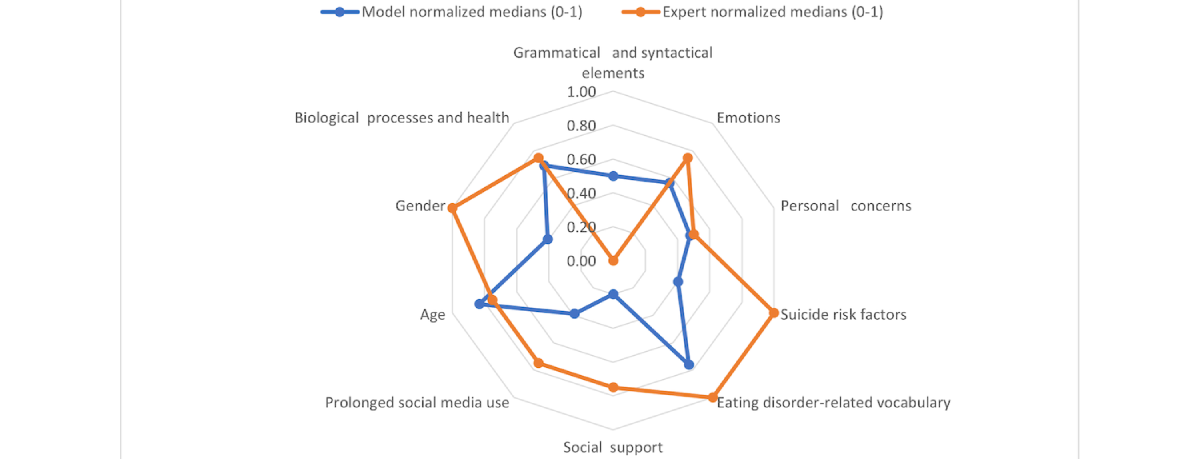
Feature types relevant for the generic model versus those relevant for the clinicians.

When comparing the feature types that were relevant according to the RFE method applied to the generic model and those that were relevant for experts (Table 4 and Figure 5), we can observe that, for the predictive model, the most relevant feature types were age, eating disorder–related vocabulary, and biological processes and health. Note that the model and clinicians agreed on the fact that eating disorder–related vocabulary was relevant, whereas clinicians also assigned a high relevance to suicide risk factors and gender. The feature types that the model considered to be less relevant were social support and prolonged social media use, whereas clinicians considered grammatical and syntactical elements less relevant.

The reason why suicide risk factors seemed to be less relevant for the model was because they are given by lexicons with a limited number of keywords, which do not necessarily always capture the existence of a given risk factor as it cannot always be explicitly described in the text. Clinicians, in contrast, are capable of identifying suicide risk factors that are described implicitly in the text and handle a wide vocabulary in comparison with the model.

As described by Ramírez-Cifuentes et al [5], the collected data set was annotated by up to 5 human experts, and the final label was determined based on the agreement of at least 3 annotators. For this analysis, the assigned labels were simplified into 2 classes: control and anorexia, with doubtful cases assigned to control.

Following the procedure described by Shing et al [48], we evaluated the performance of the individual human labelers with respect to the obtained ground truth in terms of bAcc and FNR ratios between female and male samples.

**Figure 6:**
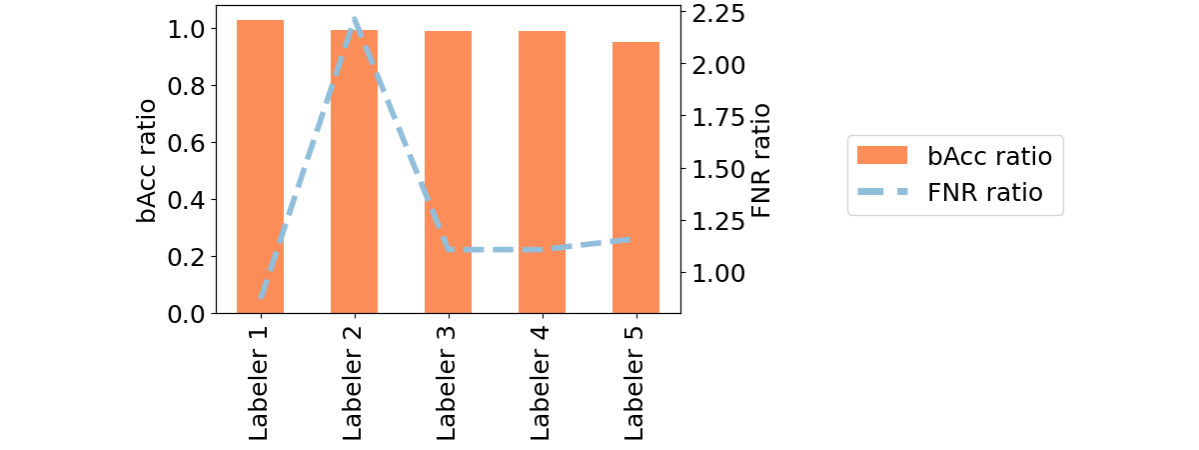
Labeler performance with respect to obtained ground truth. bAcc: balanced accuracy; FNR: false negative rate.

As shown in Figure 6, the obtained results show that labelers had greater performance for male samples, with higher accuracy and lower FNR. The only exception among the 5 experts was labeler 1, who showed higher accuracy for female samples and higher FNR for male samples. In addition, we evaluated the Cohen κ agreement between each pair of labelers for female and male samples separately, with an average of 0.807 versus 0.841, respectively, which could suggest that diagnosing male samples could be easier for human experts.

Interestingly, we see that most of the labelers had better performance when detecting AN in web-based traces. However, female samples tended to be easier to diagnose than male samples [49] during in-person consultations.

Understanding the performance of the annotators as an upper bound of the performance that an autonomous system can have for this data set, we observed that annotators had a certain level of bias (quantified in terms of performance differences across genders) that afterward seemed to be not mitigated, if not amplified, by the addition of the ML systems.

In addition to Feature Elimination, we studied 2 alternative feature selection approaches: SHAP (Shapley Additive Explanations) [50] and mutual information (MI) [51]. SHAP is a game theoretic approach used to interpret the output of ML models. It links optimal credit allocation with local explanations using Shapley values from game theory and their related extensions. On the other hand, MI is a measure between 2 (possibly multidimensional) random variables that quantifies the amount of information obtained about 1 random variable, through the other random variable (ie, dependency). It equals zero if and only if 2 random variables are independent, and higher values mean higher dependency. In the case of feature selection, the goal is to maximize the MI between the subset of selected features and the target variable.

We conducted a series of experiments in which we evaluated and compared RFE with SHAP and MI. As observed in Multimedia Appendix 1 performance disparities consistently arise between the different feature selection methodologies. The same classifiers (logistic regression) were trained with an increasing top-k number of features selected for each gender through each of the feature selection methods. It should be noted that both train and test sets included examples of both genders, and the performance is calculated with a classifier trained with the most important features for each gender. The results were calculated using 5-fold stratified cross-validation.

The obtained results are depicted in Multimedia Appendix 1 and show how the disparities in predictive performance (accuracy) between genders are consistent regardless of the feature importance selection technique that is used. Predictive performance is systematically higher for males in all the cases.

**Table 4:** Model versus expert feature type rankings.

Feature type	Model normalized median (0-1)	Model feature type ranking	Expert normalized median (0-1)	Expert feature type ranking
Age	0.83	1	0.75	2
Eating disorder–related vocabulary	0.76	2	1.00	1
Biological processes and health	0.69	3	0.75	2
Emotions	0.57	4	0.75	2
Grammatical and syntactical elements	0.50	5	0.00	4
Personal concerns	0.48	6	0.50	3
Gender	0.41	7	1.00	1
Suicide risk factors	0.40	8	1.00	1
Prolonged social media use	0.39	9	0.75	2
Social support	0.20	10	0.75	2

### Bias Mitigation (RQ 3)

#### Overview

In this section, we test the state-of-the-art techniques to obtain fair classifiers. We used the logistic regression classifier as it was the one that showed less disparities in performance while maintaining a high level of accuracy (Figure 1).

In particular, we evaluated 2 different use cases. The first one is described in the following section and corresponds to the case where the model will be used to predict whether individuals might have the disorder. We will compare the model that had better overall accuracy and the model that obtained lower FNR inequalities by gender. The best models in terms of accuracy tend to be the ones that maximize the FNR differences across genders, reducing their suitability for this use case from the point of view of algorithmic fairness. Afterward, we evaluated a second scenario where the predictive models will be used to sort the list of patients to be analyzed. First, it requires the prediction to be continuous, but in addition, for the individuals to be sorted, the predictions must have a probabilistic approach, so we know that individuals predicted with a 0.8 really have an 80% probability of being a true positive.

#### Training Fair Classifiers

The results were calculated using 5-fold cross-validation, splitting the data between training, validation, and testing. Methods that required a validation set for adjusting parameters used the validation set, and all of them were evaluated on the test set. The results reported in Table 5 correspond to the average of the 5 executions.

Tables 5 and 6 show the results of applying the bias mitigation algorithms. The former shows the results decomposed by gender, whereas the latter shows the results aggregated in ratios. On them, it can be observed that all methods lead to better bAcc ratios than those observed with the original classifier.

As can be seen in Table 6, the best bias mitigation results regarding FNR are achieved with the reweighting preprocessing algorithm, with a slight improvement with respect to the rest (see the *Algorithmic Fairness for Detecting Mental Health Status* section for a brief explanation of these algorithms). However, the improvement in terms of the FNR ratio is achieved at the cost of increasing the FNR and reducing accuracy for male samples (as shown in Table 5).

The results showed that, even when disparities can be mitigated with most of the algorithms, they cannot be eliminated. In addition, some methods failed to provide results that were substantially better than those obtained using the original classifier without any transformation.

**Table 5:** Obtained performance in terms of average balanced accuracy, average false negative rate (FNR), and average F1-score values across genders for each mitigation technique.

Technique	Female sample accuracy, mean (SD)	Male sample accuracy, mean (SD)	Female sample FNR	Male sample FNR	Female sample *F*_1_-score	Male sample *F*_1_-score
Original classifier (LogReg^a^)	0.793 (0.06)	0.948 (0.02)	0.136	0.012	0.820	0.968
Disparate impact remover	0.793 (0.03)	0.941 (0.01)	0.154	0.017	0.816	0.963
Reweighting	0.797 (0.03)	0.932 (0.03)	0.111	0.040	0.827	0.956
Calibrated equalized odds	0.793 (0.05)	0.948 (0.02)	0.136	0.012	0.820	0.968

^a^LogReg: logistic regression.

**Table 6:** Obtained performance in terms of average balanced accuracy, false negative rate (FNR), and F1-score ratios across genders for each mitigation technique.

Technique	Average balanced accuracy ratio	Average FNR ratio	Average *F*_1_-score ratio
Original classifier (LogReg^a^)	0.812	4.636	0.817
Disparate impact remover	0.851	6.075	0.839
Reweighting	0.906	2.591	0.875
Calibrated equalized odds	0.844	4.636	0.817

^a^LogReg: logistic regression.

#### Training Calibrated Classifiers

In this section, we analyze and compare the initial calibration of the different ML models trained in our experiments and apply state-of-the-art calibrators to obtain properly calibrated models after postprocessing their outputs.

**Figure 7:**
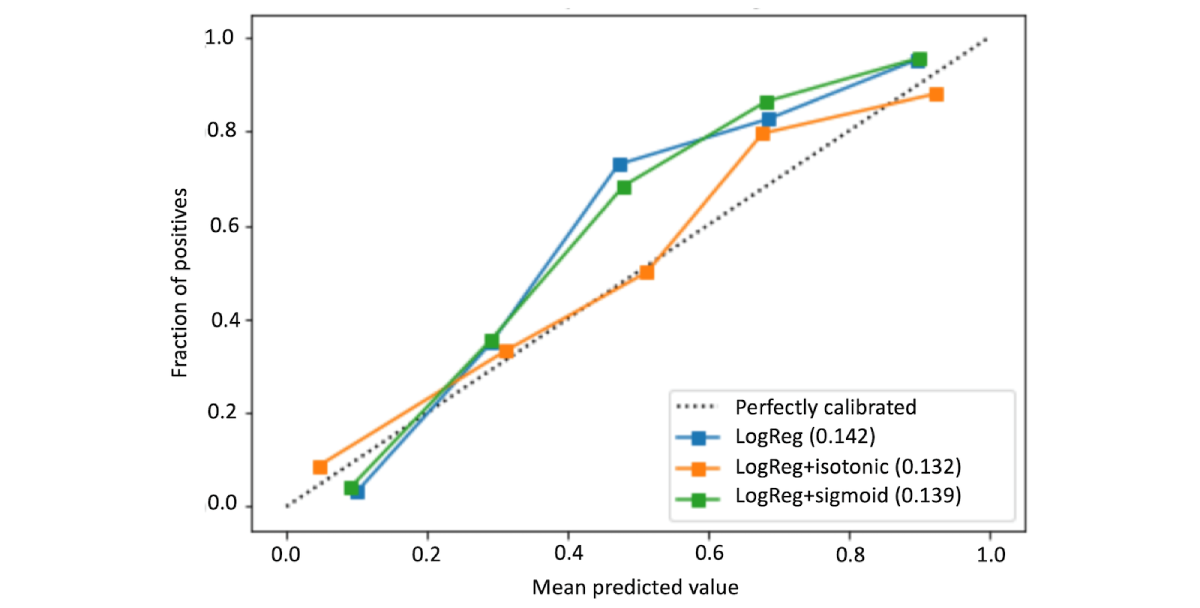
Calibration curves obtained for the original model and calibrators. LogReg: logistic regression.

The calibration results depicted in Figure 7 show that the isotonic calibrator obtained a calibration curve that was generally closer to the objective function (dotted line) for most of the thresholds.

The decision of which option is more suitable for production depends on the concrete application. Selecting the threshold that is more suitable for a given use case is generally done based on economic reasons, as occurs in many other public policy problems [52]. The following paragraphs discuss different imaginary scenarios and highlight how certain models are preferable to others depending on the characteristics of the context in which they would be deployed.

As an example of a calibration problem, suppose a scenario where all the samples predicted with a value greater than a certain threshold would be treated in a medical consultation by a practitioner. Consider a population of 100 individuals, a total budget of US $1000, and a cost of US $33 for each treated individual. This would allow up to 30 patients (30% of the population) to be considered for diagnosis in a medical consultation. In such a case, we would use a threshold of 0.7 so that only individuals with a prediction score of >0.7 were considered.

Using a perfectly calibrated model, we would expect 21 true positives (70% of the 30 individuals considered for diagnosis). In this case, given that the calibration curves depicted in Figure 7 show very similar calibration values for all the calibrators at such a threshold, there is not a strong preference for using one over the others.

Nevertheless, if we consider the same scenario but with a total budget of US $1650, we would be able to check up to 50% of the population. Therefore, we would use the 0.5 threshold, where the logistic regression classifier with the isotonic calibrator shows a clearly better performance, making it the preferred choice. Using such a classifier, we would be able to detect up to 25 true positives among the 50 individuals selected for medical consultation.

From these results, we conclude that, even when the logistic regression with the isotonic calibrator is better calibrated in general, certain use cases would make other options more or equally preferable.

## Discussion

### Principal Findings

Finally, social media offers a new space for mental health assessment. The explosive use of such platforms, especially by young people, has raised questions about the potential negative impact of these applications on the mental health of the most vulnerable. In particular, users with AN represent a very tight web-based community that has even created its own vocabulary to be identified among themselves with the goal of exchanging very unhealthy tips. In this regard, new research has been conducted on automatically detecting users at risk by analyzing their web-based activity using AI. These studies usually work on data sets that contain data from mostly female users. Thus, we would expect that these algorithms would perform better when classifying female samples than when classifying male samples as they are provided with more female instances.

Our results show that an ML model trained on the collected data exhibits relevant biases in the form of a higher FNR (fraction of false negatives among the number of positives) for female users if compared with the performance obtained for male users (RQ 1). Although the data set considered in our experiments contained a higher proportion of female examples, the model was not able to accurately separate the positive from the negative examples equally well for both genders. In particular, the overall performance (accuracy) obtained for male samples was substantially higher (approximately 10%). This is contrary to the fact that male cases tend to be more difficult to diagnose in practice [49].

We later experimented on characterizing this bias (RQ 2) by analyzing the most relevant features selected by our models for assessing female and male users separately and comparing these features with those selected by clinicians when assessing the risk of AN just based on the writings of the users. We could attest that biological processes and suicide risk factors were the key for good precision in classifying positive AN cases in male samples and that age, emotions, and personal concerns were more relevant for female samples, probably because they tend to express their feelings more in their posts. Given that the set of characteristics contained in the data set was mostly based on linguistic features, the root cause of this effect might rely on the fact that body dissatisfaction tends to be higher in women than in men [53]. Thus, it is expected that women will be more open and talkative about diets and food-related conversations regardless of whether they have an eating disorder. However, men usually do not talk about these concepts. This causes both positive and negative examples to contain similar distributions for certain features in the case of female samples, whereas those distributions are strictly different in the case of male samples. We could also confirm that automated models are not capable of identifying suicide risk factors that are described implicitly in the text.

Finally, we proposed several techniques for bias mitigation (RQ 3), and we could see that, even when disparities can be mitigated with the newly proposed algorithms, they cannot be eliminated. Our experiments revealed that finding a solution that removes all existing biases is a very arduous task. We conclude that the preference for one solution over another might depend on the concrete application system. As discussed in the *State of the Art* section, public policy problems often depend on economic factors [52], and we used 2 illustrative scenarios to showcase how the preference for one model or another highly depends on the economic characteristics of their context.

### Limitations

It is important to note the methodological limitations present in this study of algorithmic fairness on social media.

First, we acknowledge that the findings and conclusions obtained are limited to a specific eating disorder—AN. Although other eating disorders such as bulimia nervosa or eating disorder not otherwise specified (EDNOS) might have common symptoms with AN [54], the behavior and actions that characterize the individuals who have any of these disorders are quite different. Therefore, it is expected that the manifestation through language use on social media might be different among these disorders, and hence, so are the features that most accurately denote their presence and development. The exploration and comparison of the language expression on social media among these disorders is still an open problem [20].

Another limitation we observe with our study is related to the data set. As stated by Ramírez-Cifuentes et al [5], demographic attributes were inferred using an automatic approach. As with any computational method, this procedure is not free from error. Nonetheless, as the authors explained, the accuracy of the method was manually tested on a subsample of the data set. A macroaverage accuracy of 0.84 for all the gender groups of all the classes and a macroaverage accuracy of 0.80 for all the age groups of all the classes was achieved. For this reason, we still consider this a good approximation of the demographic attributes, which enables us to study the manifestation of bias and shed light on possible solutions.

Moreover, it should be noted that, even though the annotation process was rigorously conducted by 5 domain experts (n=3, 60% psychologists and n=2, 40% psychiatrists), it might not be completely accurate. The practitioners involved made their judgments using only the textual content of the posts. In a real-life scenario, a diagnosis is elaborated based on a combination of direct and indirect assessment instruments such as unstructured observation, specific questions regarding the manifesting symptoms, and formalized psychological tests. These elements allow practitioners to obtain a comprehensive cross-sectional characterization of a person’s mental health condition. Despite this limitation, we consider that, given the background and practical experience of the professionals involved in the annotation process, labeling errors would be minimal and would not influence the conclusions obtained for the whole sample of individuals analyzed in this study.

Finally, we should be aware that the conclusions drawn from the data are limited in scope to individuals who use social media, meaning that it is probably a younger and more technologically literate sample than the population as a whole [55]. Moreover, our study only included users who were active on Twitter and who chose to make their tweets publicly available. Therefore, the fairness assessment considering users from other social media platforms and even people with AN who do not have accounts on any social media platforms is out of our reach.

As future work, we will test the validity of the obtained fair models in other platforms, contexts, regions, and mental health disorders such as suicidal ideation.

### Conclusions

Web-based assessment of mental health issues using automated methods needs more attention. Most of the state-of-the-art works in this regard just measure global precision metrics without making any effort to detect gender bias. Fairness should be considered when deploying automated systems to avoid wrong assessments of mental health diagnoses when assisting clinician decisions.

## Appendix

Multimedia Appendix 1 Performance comparison across different feature selection techniques. Dashed lines indicate the obtained performance with the most important features for females and solid lanes depict performance for the most relevant features for males.

## Abbreviations

AI: artificial intelligence
AN: anorexia nervosa
bAcc: balanced accuracy
COMPAS: Correctional Offender Management Profiling for Alternative Sanctions
EDNOS: eating disorder not otherwise specified
FNR: false negative rate
GloVe: Global Vector for Word Representation
ICC: intraclass correlation coefficient
MI: mutual information
ML: machine learning
MLP: multilayer perceptron
RFE: recursive feature elimination
RQ: research question
SHAP: Shapley Additive Explanations
SVM: support vector machine
