# Supplementary material for: Gender Bias When Using Artificial Intelligence to Assess Anorexia Nervosa on Social Media: Data-Driven Study
Source: J Med Internet Res. 2023 Jun 8;25:e45184. doi: 10.2196/45184 (PMC10288345; doi:10.2196/45184)
Supplement: Multimedia Appendix 1 [file jmir_v25i1e45184_app1.docx]

As described in Section 2.4, we separated the instances by gender and applied Recursive Feature Elimination (RFE) [34] in order to analyze the relevance of features depending on the gender of the users. In addition, we studied two alternative feature selection approaches: SHAP (SHapley Additive exPlanations) [54] and Mutual Information (MI) [55]. SHAP is a game theoretic approach used to interpret the output of ML models. It links optimal credit allocation with local explanations using Shapley values from game theory and their related extensions. On the other hand, (MI) is a measure between two (possibly multi-dimensional) random variables that quantifies the amount of information obtained about one random variable, through the other random variable (i.e., dependency). It equals zero if and only if two random variables are independent, and higher values mean higher dependency. In the case of feature selection, the goal is to maximize the MI between the subset of selected features and the target variable.

We conducted a series of experiments in which we evaluated and compared RFE with SHAP and MI. As observed in Figure S1 performance disparities consistently arise between the different feature selection methodologies. The same classifiers (LogReg) were trained with an increasing top-k number of features selected for each gender through each of the feature selection methods. It should be noted that both train and test sets included examples of both genders, and the performance is calculated with a classifier trained with the most important features for each gender. The results were calculated using 5-fold stratified cross validation.


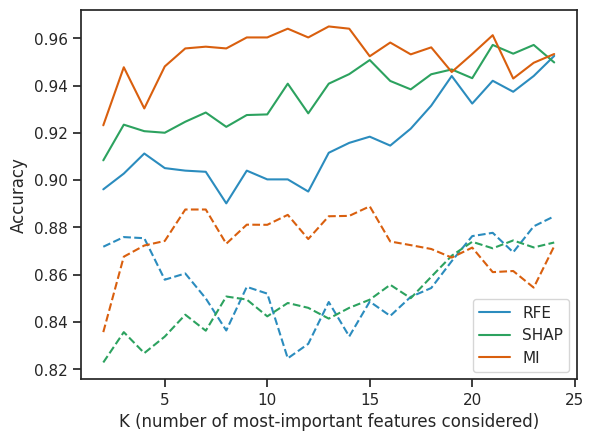


**Figure S1: Performance comparison across different feature selection techniques. Dashed lines indicate the obtained performance with most important features for females and solid lanes depict performance for most relevant features for males.**

The results show how the disparities in predictive performance (accuracy) between genders are consistent regardless of the feature importance selection technique that is utilized. Predictive performance is systematically higher for males in all the cases.
